# (*E*)-4-(4-Meth­oxy­phen­yl)but-3-en-2-one

**DOI:** 10.1107/S1600536812012214

**Published:** 2012-03-24

**Authors:** Ambika Sambyal, Manpreet Kour, Sumati Anthal, R. K. Bamzai, Rajni Kant, Vivek K. Gupta

**Affiliations:** a Post-Graduate Department of Chemistry, University of Jammu, Jammu Tawi 180 006, India; b Post-Graduate Department of Physics & Electronics, University of Jammu, Jammu Tawi 180 006, India

## Abstract

In the title compound, C_11_H_12_O_2_, the dihedral angle between the benzene ring and the but-3-en-2-one group is 4.04 (5)°. The crystal packing features chains, parallel to [-101], composed of dimers connected by weak C—H⋯O inter­actions..

## Related literature

For related structures, see: Jasinski *et al.* (2010 ▶); Fun *et al.* (2011 ▶); Dutkiewicz *et al.* (2011 ▶). For bond-length data, see: Allen *et al.* (1987 ▶).
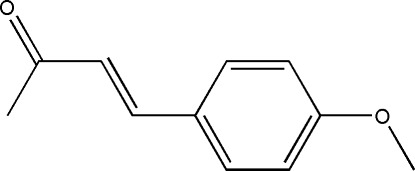


## Experimental

### 

#### Crystal data

C_11_H_12_O_2_
*M* *_r_* = 176.21
Monoclinic, 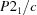

*a* = 10.1623 (19) Å
*b* = 13.292 (3) Å
*c* = 6.6488 (13) Å
β = 98.486 (3)°
*V* = 888.3 (3) Å^3^
*Z* = 4
Mo *K*α radiation
μ = 0.09 mm^−1^
*T* = 100 K
0.30 × 0.30 × 0.10 mm

#### Data collection

Bruker APEXII CCD diffractometer
Absorption correction: multi-scan (*SADABS*; Bruker, 2009 ▶) *T*_min_ = 0.920, *T*_max_ = 0.980
4492 measured reflections
1556 independent reflections
1332 reflections with *I* > 2σ(*I*)
*R*_int_ = 0.057

#### Refinement

*R*[*F* ^2^ > 2σ(*F* ^2^)] = 0.063
*wR*(*F* ^2^) = 0.150
*S* = 1.10
1556 reflections
121 parameters
H-atom parameters constrained
Δρ_max_ = 0.36 e Å^−3^
Δρ_min_ = −0.60 e Å^−3^

### 

Data collection: *APEX2* (Bruker, 2009 ▶); cell refinement: *SAINT* (Bruker, 2009 ▶); data reduction: *SAINT*; program(s) used to solve structure: *SHELXS97* (Sheldrick, 2008 ▶); program(s) used to refine structure: *SHELXL97* (Sheldrick, 2008 ▶); molecular graphics: *ORTEP-3 for Windows* (Farrugia, 1997 ▶); software used to prepare material for publication: *PLATON* (Spek, 2009 ▶).

## Supplementary Material

Crystal structure: contains datablock(s) I, global. DOI: 10.1107/S1600536812012214/gk2469sup1.cif

Structure factors: contains datablock(s) I. DOI: 10.1107/S1600536812012214/gk2469Isup2.hkl

Supplementary material file. DOI: 10.1107/S1600536812012214/gk2469Isup3.cml

Additional supplementary materials:  crystallographic information; 3D view; checkCIF report

## Figures and Tables

**Table 1:** Hydrogen-bond geometry (Å, °)

*D*—H⋯*A*	*D*—H	H⋯*A*	*D*⋯*A*	*D*—H⋯*A*
C6′—H6′⋯O2^i^	0.93	2.52	3.296 (2)	141
C6—H6*B*⋯O2^ii^	0.96	2.57	3.533 (2)	176

Symmetry codes: (i) 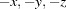; (ii) 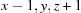.

**Fig. 1.**
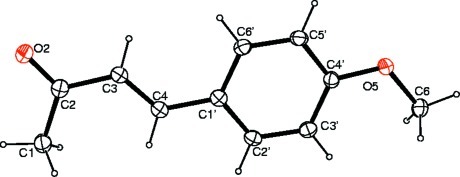
ORTEP view of the molecule with the atom-labeling scheme. The displacement ellipsoids are drawn at the 40% probability level. H atoms are shown as small spheres of arbitrary radii.

**Fig. 2.**
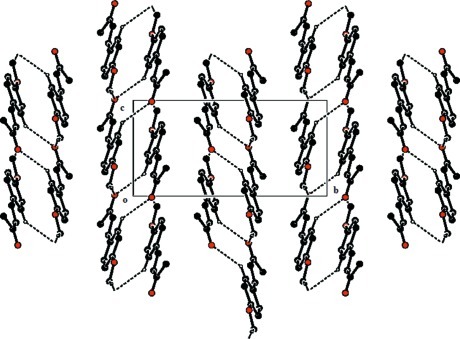
The packing arrangement of molecules viewed down the a axis. Hydrogen bonds are shown with dashed lines.

## supplementary crystallographic information

### Comment

The title compound belongs to the class of unsaturated α,β- acyclic ketones. α,β- unsaturated ketones have been found useful in the preparation of a wide variety of nitrogen heterocycles both in solution phase and solid state. Many of these synthesis have proceeded through interesting mechanisms. These compounds have been used as substrates for the preparation of anti-cancer, cell-specific triarylpyridines *via* immobilized bismuth nitrate catalyzed cascade reactions. These compounds have been extensively used in the preparation of cardiovascular Hantzsch products, many of which are prescribed drugs. α,β- unsaturated ketones are easily elaborated to anti-anxiety diazepines which also regulate our central nervous system. When treated with hydrazine, α,β- unsaturated compounds yield substituted pyrazoles which have a wide spectrum of bioactivity. The molecular structure of the title compound is shown in Fig.1. The bond distances are within normal ranges (Allen *et al.*, 1987) and comparable to those in related structures (Jasinski *et al.*, 2010; Fun *et al.*, 2011; Dutkiewicz *et al.*, 2011). The six bond lengths in the benzene ring lie in the range 1.355 (2)–1.391 (2) Å with an average value of 1.372 (2) Å. The average bond angle of the phenyl ring is 120.0 (1)°. In the title compound the benzene ring is perfectly planar with a maximum deviation of 0.006 (2) Å for C2'. The dihedral angle between the benzene ring and the acyclic chain is 4.04 (5)°. In the crystal structure, intermolecular C—H···O hydrogen bonds link the molecules into chains (Fig.2).

### Experimental

Normally, α,β-unsaturated compounds are prepared by the reaction of an aldehyde and an active methylene compounds by Claisen-Schmidt reaction, using a strong base or an acid as catalyst. Under these reaction conditions, aromatic aldehydes and acetone react hard to form diarylidene ketone by double Claisen condensation. Monocondensation processes are known but yields are poor. To improve yield of monocondensation products, a search for catalyst was undertaken and sodium tungstate in ethanol was found as the catalyst of choice. The title compound was prepared in 96% yield by stirring the mixture of anisaldehyde (1 X 10-2 mol) and acetone (1 X 10-2 mol) in the presence of sodium tungstate (30 mol %) using ethanol as solvent at room temperature (25°C) for 24h. The reaction was monitored by thin layer chromatography. On completion of the reaction, the reaction mixture was diluted with water (25ml) and extracted with ethyl acetate (50 ml). The organic layer was washed with water, brine and water, dried over anhydrous sodium sulphate and concentrated. The title compound was purified by column chromatography on silica gel, using CH_2_Cl_2_-EtOAC (9:1v/v) as solvent. The compound was crystallized from chloroform-methanol, m.p. 445K. Single crystals for XRD study were obtained by slow evaporation of chloroform solution.

### Refinement

All H atoms were positioned geometrically and were treated as riding on their parent C atoms, with C—H distances of 0.93–0.96 Å and with *U*_iso_(H) = 1.2Ueq(C), except for the methyl groups where *U*_iso_(H) = 1.5Ueq(C),.

### Crystal data

**Table:**

C_11_H_12_O_2_	*F*(000) = 376
*M**_r_* = 176.21	*D*_x_ = 1.318 Mg m^−^^3^
Monoclinic, *P*2_1_/*c*	Mo *K*α radiation, λ = 0.71073 Å
Hall symbol: -P 2ybc	Cell parameters from 2041 reflections
*a* = 10.1623 (19) Å	θ = 2.5–28.1°
*b* = 13.292 (3) Å	µ = 0.09 mm^−^^1^
*c* = 6.6488 (13) Å	*T* = 100 K
β = 98.486 (3)°	Hexagonal plate, colourless
*V* = 888.3 (3) Å^3^	0.30 × 0.30 × 0.10 mm
*Z* = 4	

### Data collection

**Table:**

Bruker APEXII CCD diffractometer	1556 independent reflections
Radiation source: fine-focus sealed tube	1332 reflections with *I* > 2σ(*I*)
Graphite monochromator	*R*_int_ = 0.057
Detector resolution: 0 pixels mm^-1^	θ_max_ = 25.0°, θ_min_ = 2.5°
phi and ω scans	*h* = −11→12
Absorption correction: multi-scan (*SADABS*; Bruker, 2009)	*k* = −15→14
*T*_min_ = 0.920, *T*_max_ = 0.980	*l* = −7→7
4492 measured reflections	

### Refinement

**Table:**

Refinement on *F*^2^	Secondary atom site location: difference Fourier map
Least-squares matrix: full	Hydrogen site location: inferred from neighbouring sites
*R*[*F*^2^ > 2σ(*F*^2^)] = 0.063	H-atom parameters constrained
*wR*(*F*^2^) = 0.150	*w* = 1/[σ^2^(*F*_o_^2^) + (0.0943*P*)^2^ + 0.0951*P*] where *P* = (*F*_o_^2^ + 2*F*_c_^2^)/3
*S* = 1.10	(Δ/σ)_max_ = 0.001
1556 reflections	Δρ_max_ = 0.36 e Å^−^^3^
121 parameters	Δρ_min_ = −0.60 e Å^−^^3^
0 restraints	Extinction correction: *SHELXL97* (Sheldrick, 2008), Fc^*^=kFc[1+0.001xFc^2^λ^3^/sin(2θ)]^-1/4^
Primary atom site location: structure-invariant direct methods	Extinction coefficient: 0.093 (13)

### Special details

**Table:**

Geometry. All esds (except the esd in the dihedral angle between two l.s. planes) are estimated using the full covariance matrix. The cell esds are taken into account individually in the estimation of esds in distances, angles and torsion angles; correlations between esds in cell parameters are only used when they are defined by crystal symmetry. An approximate (isotropic) treatment of cell esds is used for estimating esds involving l.s. planes.
Refinement. Refinement of F^2^ against ALL reflections. The weighted R-factor wR and goodness of fit S are based on F^2^, conventional R-factors R are based on F, with F set to zero for negative F^2^. The threshold expression of F^2^ > 2sigma(F^2^) is used only for calculating R-factors(gt) etc. and is not relevant to the choice of reflections for refinement. R-factors based on F^2^ are statistically about twice as large as those based on F, and R- factors based on ALL data will be even larger.

### Fractional atomic coordinates and isotropic or equivalent isotropic displacement parameters (Å^2^)

**Table:**

	*x*	*y*	*z*	*U*_iso_*/*U*_eq_	
C1	0.32731 (14)	0.16937 (12)	0.2922 (2)	0.0270 (4)	
H1A	0.4029	0.1751	0.2218	0.040*	
H1B	0.3505	0.1293	0.4123	0.040*	
H1C	0.3005	0.2352	0.3298	0.040*	
O2	0.23069 (10)	0.09565 (8)	−0.01128 (16)	0.0272 (4)	
C2	0.21533 (15)	0.12060 (11)	0.1565 (2)	0.0216 (4)	
C3	0.08653 (15)	0.10549 (11)	0.2238 (2)	0.0205 (4)	
H3	0.0184	0.0778	0.1318	0.025*	
C4	0.06085 (14)	0.12829 (10)	0.4045 (2)	0.0197 (4)	
H4	0.1314	0.1531	0.4961	0.024*	
O5	−0.42876 (10)	0.09838 (8)	0.69111 (15)	0.0255 (4)	
C6	−0.43869 (14)	0.12266 (12)	0.8912 (2)	0.0257 (4)	
H6A	−0.3794	0.0808	0.9809	0.039*	
H6B	−0.5284	0.1119	0.9157	0.039*	
H6C	−0.4152	0.1920	0.9155	0.039*	
C1'	−0.06661 (14)	0.11909 (10)	0.4784 (2)	0.0194 (4)	
C2'	−0.07619 (13)	0.15059 (11)	0.6694 (2)	0.0209 (4)	
H2'	−0.0009	0.1759	0.7502	0.025*	
C3'	−0.19429 (14)	0.14616 (11)	0.7475 (2)	0.0220 (4)	
H3'	−0.1984	0.1693	0.8784	0.026*	
C4'	−0.30685 (14)	0.10736 (10)	0.6309 (2)	0.0199 (4)	
C5'	−0.29933 (14)	0.07434 (11)	0.4401 (2)	0.0221 (4)	
H5'	−0.3743	0.0479	0.3605	0.027*	
C6'	−0.18091 (14)	0.07994 (11)	0.3644 (2)	0.0212 (4)	
H6'	−0.1770	0.0569	0.2333	0.025*	

### Atomic displacement parameters (Å^2^)

**Table:**

	*U*^11^	*U*^22^	*U*^33^	*U*^12^	*U*^13^	*U*^23^
C1	0.0235 (8)	0.0307 (8)	0.0275 (9)	−0.0024 (6)	0.0064 (6)	−0.0031 (6)
O2	0.0258 (7)	0.0343 (7)	0.0224 (7)	−0.0017 (4)	0.0069 (5)	−0.0039 (4)
C2	0.0237 (8)	0.0181 (7)	0.0232 (9)	0.0031 (6)	0.0047 (6)	0.0033 (6)
C3	0.0185 (8)	0.0207 (8)	0.0217 (9)	−0.0005 (5)	0.0012 (6)	0.0005 (5)
C4	0.0193 (8)	0.0163 (7)	0.0229 (9)	0.0002 (5)	0.0010 (6)	0.0018 (6)
O5	0.0179 (6)	0.0374 (7)	0.0217 (7)	−0.0014 (4)	0.0039 (4)	−0.0016 (4)
C6	0.0222 (8)	0.0321 (9)	0.0241 (9)	0.0019 (6)	0.0077 (6)	−0.0007 (7)
C1'	0.0206 (8)	0.0153 (7)	0.0220 (8)	0.0018 (5)	0.0021 (6)	0.0027 (6)
C2'	0.0198 (8)	0.0189 (8)	0.0227 (9)	−0.0015 (6)	−0.0006 (6)	0.0003 (6)
C3'	0.0250 (8)	0.0205 (8)	0.0201 (8)	0.0009 (6)	0.0021 (6)	−0.0006 (6)
C4'	0.0181 (8)	0.0207 (8)	0.0210 (9)	0.0023 (6)	0.0035 (6)	0.0030 (6)
C5'	0.0209 (7)	0.0247 (8)	0.0194 (8)	−0.0007 (6)	−0.0012 (6)	0.0006 (6)
C6'	0.0229 (8)	0.0226 (8)	0.0177 (8)	0.0016 (6)	0.0015 (6)	0.0006 (6)

### Geometric parameters (Å, º)

**Table:**

C1—C2	1.492 (2)	C6—H6B	0.9600
C1—H1A	0.9600	C6—H6C	0.9600
C1—H1B	0.9600	C1'—C2'	1.355 (2)
C1—H1C	0.9600	C1'—C6'	1.391 (2)
O2—C2	1.1959 (18)	C2'—C3'	1.377 (2)
C2—C3	1.458 (2)	C2'—H2'	0.9300
C3—C4	1.302 (2)	C3'—C4'	1.383 (2)
C3—H3	0.9300	C3'—H3'	0.9300
C4—C1'	1.4568 (19)	C4'—C5'	1.355 (2)
C4—H4	0.9300	C5'—C6'	1.373 (2)
O5—C4'	1.3622 (16)	C5'—H5'	0.9300
O5—C6	1.3872 (18)	C6'—H6'	0.9300
C6—H6A	0.9600		
			
C2—C1—H1A	109.5	H6A—C6—H6C	109.5
C2—C1—H1B	109.5	H6B—C6—H6C	109.5
H1A—C1—H1B	109.5	C2'—C1'—C6'	117.18 (13)
C2—C1—H1C	109.5	C2'—C1'—C4	118.74 (13)
H1A—C1—H1C	109.5	C6'—C1'—C4	124.08 (14)
H1B—C1—H1C	109.5	C1'—C2'—C3'	121.82 (13)
O2—C2—C3	119.65 (14)	C1'—C2'—H2'	119.1
O2—C2—C1	119.45 (13)	C3'—C2'—H2'	119.1
C3—C2—C1	120.87 (13)	C2'—C3'—C4'	119.97 (14)
C4—C3—C2	124.22 (14)	C2'—C3'—H3'	120.0
C4—C3—H3	117.9	C4'—C3'—H3'	120.0
C2—C3—H3	117.9	C5'—C4'—O5	115.21 (13)
C3—C4—C1'	126.94 (14)	C5'—C4'—C3'	119.27 (13)
C3—C4—H4	116.5	O5—C4'—C3'	125.52 (13)
C1'—C4—H4	116.5	C4'—C5'—C6'	119.95 (13)
C4'—O5—C6	117.34 (11)	C4'—C5'—H5'	120.0
O5—C6—H6A	109.5	C6'—C5'—H5'	120.0
O5—C6—H6B	109.5	C5'—C6'—C1'	121.81 (14)
H6A—C6—H6B	109.5	C5'—C6'—H6'	119.1
O5—C6—H6C	109.5	C1'—C6'—H6'	119.1
			
O2—C2—C3—C4	178.60 (14)	C6—O5—C4'—C3'	−5.4 (2)
C1—C2—C3—C4	−3.1 (2)	C2'—C3'—C4'—C5'	−0.2 (2)
C2—C3—C4—C1'	177.22 (12)	C2'—C3'—C4'—O5	179.55 (12)
C3—C4—C1'—C2'	−177.26 (15)	O5—C4'—C5'—C6'	−179.98 (12)
C3—C4—C1'—C6'	2.5 (2)	C3'—C4'—C5'—C6'	−0.2 (2)
C6'—C1'—C2'—C3'	−1.2 (2)	C4'—C5'—C6'—C1'	−0.1 (2)
C4—C1'—C2'—C3'	178.55 (12)	C2'—C1'—C6'—C5'	0.8 (2)
C1'—C2'—C3'—C4'	1.0 (2)	C4—C1'—C6'—C5'	−178.96 (12)
C6—O5—C4'—C5'	174.34 (13)		

### Hydrogen-bond geometry (Å, º)

**Table:**

*D*—H···*A*	*D*—H	H···*A*	*D*···*A*	*D*—H···*A*
C6′—H6′···O2^i^	0.93	2.52	3.296 (2)	141
C6—H6*B*···O2^ii^	0.96	2.57	3.533 (2)	176

Symmetry codes: (i) −*x*, −*y*, −*z*; (ii) *x*−1, *y*, *z*+1.
